# Antibodies against recombinant human alpha-glucosidase do not seem to affect clinical outcome in childhood onset Pompe disease

**DOI:** 10.1186/s13023-022-02175-2

**Published:** 2022-02-02

**Authors:** Harmke A. van Kooten, Imke A. M. Ditters, Marianne Hoogeveen-Westerveld, Edwin H. Jacobs, Johanna M. P. van den Hout, Pieter A. van Doorn, W. W. M. Pim Pijnappel, Ans T. van der Ploeg, Nadine A. M. E. van der Beek

**Affiliations:** 1 Department of Neurology, Center for Lysosomal and Metabolic Diseases, Erasmus MC, University Medical Center Rotterdam, Rotterdam, The Netherlands; 2 Department of Pediatrics, Center for Lysosomal and Metabolic Diseases, Erasmus MC - Sophia Children’s Hospital, University Medical Center Rotterdam, Rotterdam, The Netherlands; 3 Department of Pediatrics, Department of Clinical Genetics, Center for Lysosomal and Metabolic Diseases, Erasmus MC, University Medical Center, Rotterdam, The Netherlands; 4 Department of Neurology, Erasmus University Medical Center, Mailbox 2040, 3000 CA Rotterdam, the Netherlands

**Keywords:** Antibodies, Enzyme replacement therapy, Pompe disease, Recombinant human alpha-glucosidase

## Abstract

**Background:**

Enzyme replacement therapy (ERT) with recombinant human alpha-glucosidase (rhGAA, alglucosidase alfa) has improved survival, motor outcomes, daily life activity and quality of life in Pompe patients. However, ERT in Pompe disease often induces formation of antibodies, which may reduce the efficacy of treatment and can lead to adverse events. In this study antibody formation and their effect on clinical outcome in patients with childhood onset Pompe disease treated with enzyme replacement therapy (ERT) with recombinant human alpha-glucosidase (rhGAA) are analyzed.

**Methods:**

Enzyme-linked immunosorbent assay (ELISA) was used to determine anti-rhGAA antibody titers at predefined time points. The effect of antibodies on rhGAA activity (neutralizing effects) was measured in vitro. Clinical effects were evaluated by assessing muscle strength (MRC score) and function (QMFT-score), pulmonary function and infusion associated reactions (IARs).

**Results:**

Twenty-two patients were included (age at start ERT 1.1–16.4 years, median treatment duration 12.4 years). Peak antibody titers were low (< 1:1250) in 9%, intermediate (1:1250–1:31,250) in 68% and high (≥ 1:31250) in 23% of patients; three patients (14%) had more than one titer of ≥ 1:31,250. Four patients (18%) experienced IARs; two patients from the high titer group had 86% of all IARs. Inhibition of intracellular GAA activity (58%) in vitro was found in one sample. The clinical course did not appear to be influenced by antibody titers.

**Conclusions:**

Ninety-one percent of childhood onset Pompe patients developed anti-rhGAA antibodies (above background level), a minority of whom had high antibody titers at repeated time points, which do not seem to interfere with clinical outcome. High antibody titers may be associated with the occurrence of IARs. Although the majority of patients does not develop high titers; antibody titers should be determined in case of clinical deterioration.

**Supplementary Information:**

The online version contains supplementary material available at 10.1186/s13023-022-02175-2.

## Background

Pompe disease (glycogen storage disease type II or acid maltase deficiency, OMIM #232300) is a rare inherited metabolic and neuromuscular disorder, caused by disease-associated variants in the *GAA* gene, leading to deficiency of the lysosomal enzyme acid α-glucosidase [1]. In patients with the classic infantile form, a complete lack of α-glucosidase activity causes a rapidly progressive disease, characterized by a hypertrophic cardiomyopathy and severe skeletal and respiratory muscle weakness. Untreated, these infants die within the first year of life [2]. In ‘late-onset’ or ‘non-classic’ Pompe disease, which can present at any age, progressive limb-girdle and respiratory muscle weakness are the main symptoms [3, 4].

Enzyme replacement therapy (ERT) with recombinant human alpha-glucosidase (rhGAA, alglucosidase alfa, Myozyme®) received market authorization in 2006 and has improved survival, motor outcomes, daily life activity and quality of life in Pompe patients [5–10]. However, similar to any other disease treated with human recombinant proteins, ERT in Pompe disease often induces formation of antibodies, which may reduce the efficacy of treatment and can lead to adverse events [11]. Risk factors for the development of high antibody titers in classic infantile Pompe patients are the absence of cross reactive immunological material (CRIM) and an older age at start of therapy [12, 13]. In adults, antibody formation generally does not seem to interfere with ERT efficacy. However, some cases of adults with high antibody titers negatively affecting treatment outcomes have been reported [14–17]. Moreover, high antibody titers are associated with the occurrence of infusion-associated reactions (IARs) in adults [14]. Patients who developed symptoms and started ERT during childhood are an underrepresented group in previous studies on antibody formation during treatment with ERT. Here, we investigate the role of antibody formation in these patients, focusing on the antibody titers and their possible effects on clinical outcome and the occurrence of IARs during long-term treatment with ERT.

## Results

### Patient characteristics

The characteristics of the 22 patients included in this study are summarized in Table 1. Thirteen patients (58%) were male. Fifteen patients (68%) carried the common c.-32-13T > G (IVS1) disease-associated variant on one allele. The c.510C > T genetic modifier on the IVS1 allele, which is associated with lower α-glucosidase activity in fibroblasts and earlier symptom onset, was analysed in all IVS1 patients (n = 15) and found in seven patients (47%) [18]. Interestingly, five patients with the IVS1 variant had onset of symptoms within their first year of life, two of whom carried the genetic modifier. Three patients had hypertrophic cardiomyopathy at presentation (patient 3, 11 and 22; none of whom had the common IVS1 variant), which resolved after start of ERT in all three (data not shown). Age at start of ERT ranged from 1.1 to 16.4 years (median 10.3 years), the median duration of treatment was 12.4 years (range 2.2–19.6 years). Initially, patients 7 and 8 received rhGAA from transgenic rabbit milk [19]. They were switched to infusions of rhGAA derived from Chinese hamster ovarian cells after approximately 3 years, initially receiving a dose of 20 mg/kg every other week and later 30 and 40 mg/kg every other week, respectively [20]. Two patients (patient 11 and 22) were treated with 40 mg/kg every week from start of treatment, while patient 13 switched from 20 mg/kg every other week to 40 mg/kg every other week during the course of the study because of clinical deterioration. All other patients (n = 17) were treated with the standard dose of 20 mg/kg every other week during the entire study. In patient 3, ERT was discontinued after 10 years of treatment because of end stage disease, this patient deceased 7.6 months after cessation of therapy.

**Table 1:** Patient characteristics

Patient	Sex	Age symptom onset (y)	Disease-associated variant 1	Disease-associated variant 2	Age start ERT (y)	ERT duration (y)	Wheelchair use	Ventilation use	IARs	Peak antibody titer
**1**	**F**	**0.2**	**c.2481 + 102_2646 + 31del**	**c.2102T > C**	**1.7**	**5.9**			**Yes**	**1:156,250**
**2**^**§,a**^	**M**	**11.8**	**c.-32-13T > G (IVS1)**	**c.1441T > C**	**16.0**	**12.9**			**No**	**1:31,250**
**3**^**¥,†**^	**F**	**1.0**	**c.875A > G**	**Unknown**	**12.7**	**10.0**	**Yes (6y)**	**Invasive (6y)**	**Yes**	**1:31,250**
**4**	**M**	**5.0**	**c.-32-13T > G (IVS1)**	**c.525delT**	**11.0**	**9.6**			**No**	**1:31,250**
**5**	**M**	**0.8**	**c.-32-13T > G (IVS1)**	**c.525del**	**9.8**	**7.9**			**No**	**1:31,250**
*6*^*§,b*^	*M*	*2.5*	*c.-32-13T > G (IVS1)*	*c.2331 + 2T > A*	*13.0*	*13.9*			*No*	*1:6400*
*7*^*a*^	*F*	*10.0*	*c.877G > A + c.271G > A*	*c.-32-3C > A*	*16.4*	*19.6*	*Yes (16y)*	*Non-invasive (12y)*	*No*	*1:6250*
8^*,c^	*M*	*0.5*	*c.-32-13T > G (IVS1)*	*c.525del*	*11.9*	*19.3*	*Yes (11y)*		*No*	*1:6250*
*9*^*§,b*^	*F*	*6.5*	*c.-32-13T > G (IVS1)*	*c.525del*	*12.7*	*13.9*			*No*	*1:6250*
*10*	*M*	*8.9*	*c.-32-13T > G (IVS1)*	*c.525del*	*10.5*	*6.4*			*No*	*1:6250*
*11*^*¥,d*^	*M*	*1.0*	*c.379_380del*	*c.875A > G*	*3.4*	*3.4*			*No*	*1:6250*
12^*,e^	*M*	*2.0*	*c.-32-13T > G (IVS1)*	*c.525del*	*13.1*	*14.3*			*Yes*	*1:1250*
*13*^*b,f*^	*M*	*2.7*	*c.1634C > T*	*c.2481 + 102_2646 + 31del*	*6.0*	*13.9*			*No*	*1:1250*
*14*^*b*^	*M*	*1.0*	*c.-32-13T > G (IVS1)*	*c.525del*	*15.2*	*13.9*		*Non-invasive (12y)*	*No*	*1:1250*
*15*^*b*^	*F*	*0.8*	*c.-32-13T > G (IVS1)*	*c.923A > C*	*8.9*	*13.9*			*No*	*1:1250*
*16*^*§,‡,e*^	*M*	*0.8*	*c.-32-13T > G (IVS1)*	*c.2135T > C*	*2.9*	*13.7*			*Yes*	*1:1250*
*17*	*F*	*6.0*	*c.1829C > T*	*c.1912G > T*	*10.1*	*12.8*	*Yes (9y)*	*Non-invasive (8y)*	*No*	*1:1250*
*18*^*§*^	*M*	*13.0*	*c.-32-13T > G (IVS1)*	*c.1933G > A*	*14.3*	*11.9*			*No*	*1:1250*
*19*^*§,‡*^	*F*	*0.8*	*c.-32-13T > G (IVS1)*	*c.2135T > C*	*1.1*	*9.9*			*No*	*1:1250*
*20*	*F*	*5.0*	*c.-32-13T > G (IVS1)*	*c.2331 + 2T > A*	*8.5*	*8.5*			*No*	*1:1250*
***21***^***§***^	***F***	***1.0***	***c.-32-13T > G (IVS1)***	***c.525del***	***1.9***	***3.9***			***No***	***1:250***
***22***^***¥,d***^	***M***	***1.3***	***c.1115A > T***	***c.1115A > T***	***1.4***	***2.2***			***No***	***1:250***
*Overall*	Male N = 13 (58%)	1.7 (0.2–13.0)	IVS1 N = 15 (68%)		10.3 (1.1–16.4)	12.4 (2.2–19.6)	N = 4 (18%)	N = 4 (18%)	IARs N = 4 (18%)	High peak titer N = 5 (23%)

Bold = high peak titer patients, italics = intermediate peak titer patients, bold italics = low peak titer patients

Data are median (range) unless indicated otherwise. § IVS1 patients with c.510C > T genetic modifier; ¥ patients with hypertrophic cardiomyopathy at presentation; † patient deceased; * siblings; ‡ siblings; ^a^ patients who participated in van der Ploeg et al. 2010 (start ERT in 2006); ^b^ patients who participated in Capelle et al. 2010 (start ERT in 2005); ^c^patients who initially started on recombinant human alpha-glucosidase from rabbit milk in 1999 and were switched to a higher dose of alglucosidase alfa (Winkel 2004); ^d^patients treated with a dose of 40 mg/kg every week; ^e^ patients who started ERT in 2004/2005 as part of an expanded access program; ^f^ patient switched to a dose of 40 mg/kg every 2 weeks after 13.3 years of treatment

### Anti-rhGAA antibodies

**Fig. 1:**
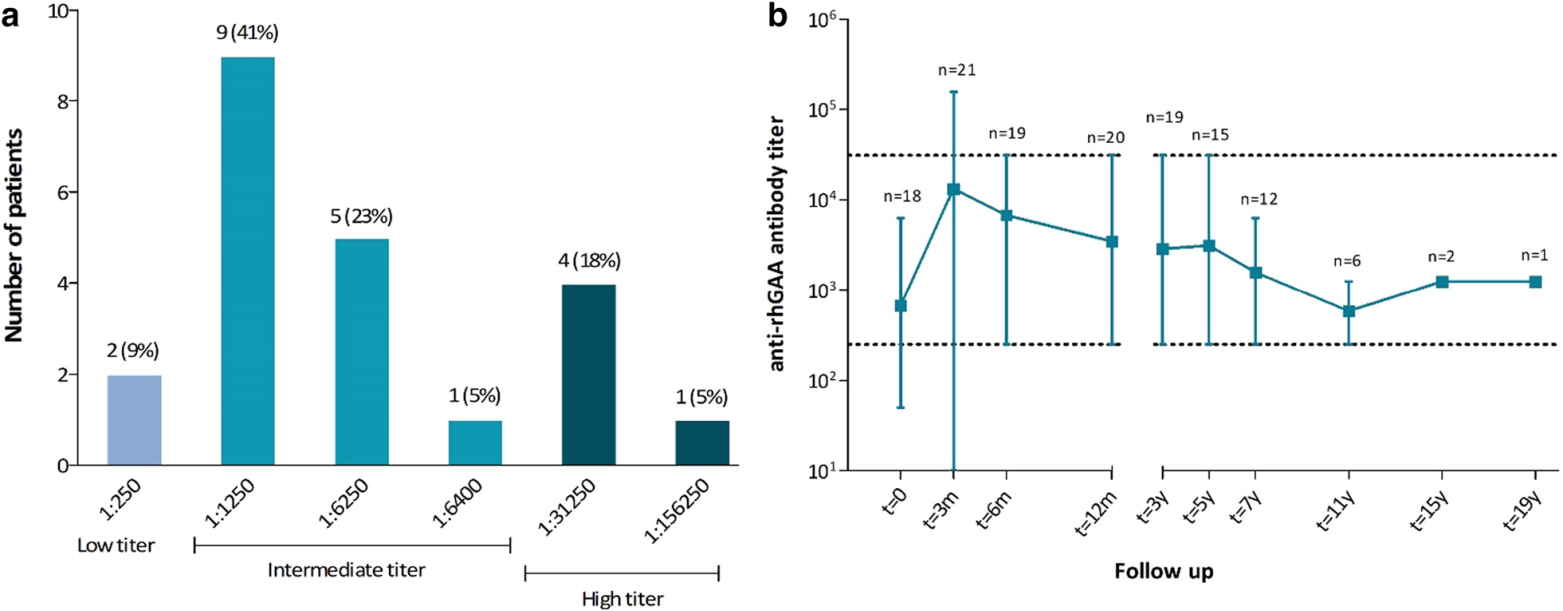
Peak anti-recombinant human acid α-glucosidase (anti-rhGAA) antibody titers. **a** Distribution of peak anti-rhGAA antibody titers, across three groups: low titer (< 1:1250), intermediate titer (1:1,250– < 1:31,25) and high titer (≥ 1:31,250). **b** Mean and range of anti-rhGAA antibody titer over time for the total group. The number of patients at each time point is indicated. The dotted lines indicate the thresholds between low-intermediate and intermediate-high titers

**Fig. 2:**
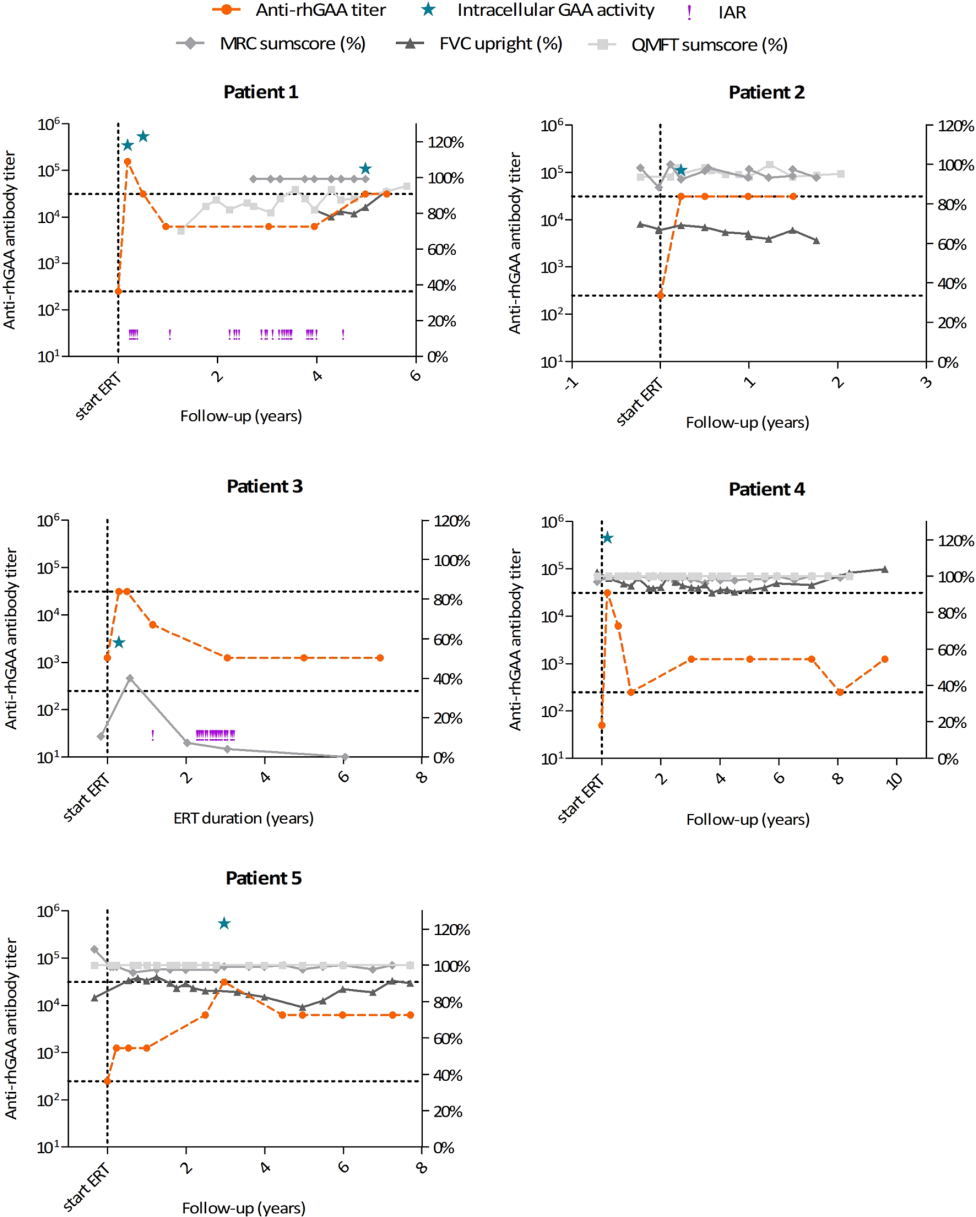
Clinical parameters, anti-recombinant human acid α-glucosidase (anti-rhGAA) antibody titers, neutralizing effects and infusion associated reactions (IARs) for five patients with a high or very high peak antibody titer. Each graph represents an individual patient. For each patient, anti-rhGAA antibody titers are shown on the left Y-axis, the effect of antibody titers on intracellular rhGAA activity (%) is shown on the right Y-axis. The occurrence of IARs is displayed (exclamation marks). Clinical outcome parameters, the Medical Research Council (MRC) sum score (expressed as a percentage of maximal muscle strength), Forced Vital Capacity (FVC) in upright position (expressed as a percentage of predicted normal values) and Quick Motor Function Test (QMFT) sum score (expressed as a percentage of the maximal score), are shown on the right Y-axis

**Fig. 3:**
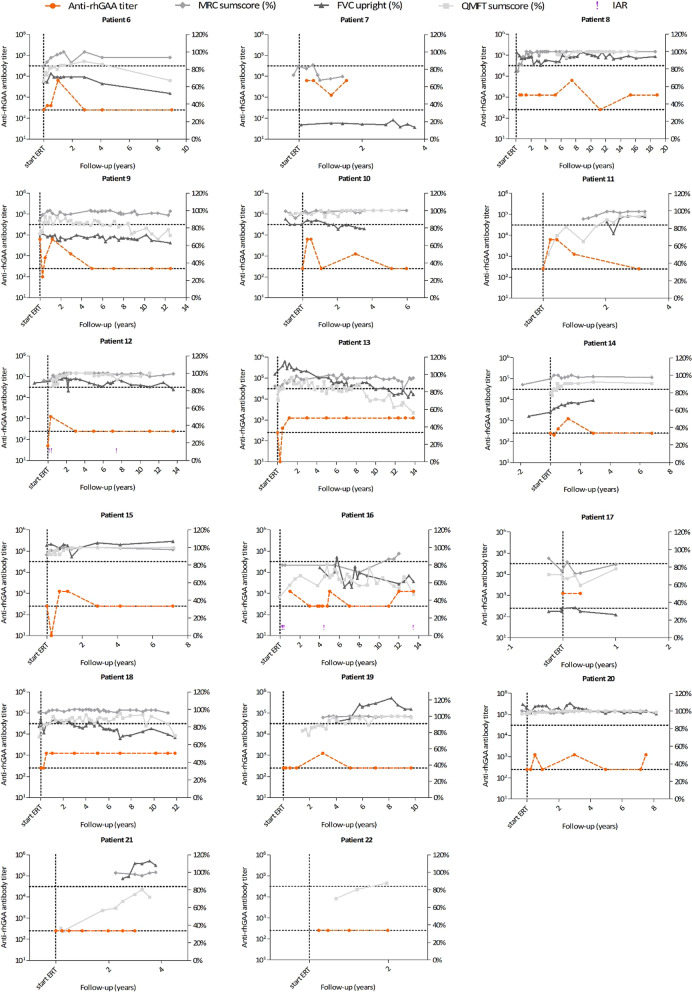
Clinical parameters, anti-recombinant human acid α-glucosidase (anti-rhGAA) antibody titers, neutralizing effects and infusion associated reactions (IARs) for patients with an intermediate (patient 6–20) or low (patient 21 and 22) peak antibody titer. Each graph represents an individual patient. For each patient, anti-rhGAA antibody titers are shown on the left Y-axis. The occurrence of IARs is displayed (exclamation marks). Clinical outcome parameters, the Medical Research Council (MRC) sum score (expressed as a percentage of maximal muscle strength), Forced Vital Capacity (FVC) in upright position (expressed as a percentage of predicted normal values) and Quick Motor Function Test (QMFT) sum score (expressed as a percentage of the maximal score) are shown on the right Y-axis

Twenty patients (91%) developed anti-rhGAA IgG antibodies above the background titer of 1:250. In total, 12 of 152 samples (8%) showed a high titer (≥ 1:31,250) in at least one of the duplicate experiments. Based on the highest titer (peak titer) observed during the study period, patients were divided into three titer groups: two patients (9%) had low titers, 15 patients (68%) developed intermediate titers and five patients (23%) developed high titers, one of whom had a very high titer (1:156,250) at one time point (Fig. 1a). The mean antibody titer at group level showed a peak in antibody titer at three months of ERT, after which titers declined (Fig. 1b). Antibody titer courses of individual patients are shown in Fig. 2 (high titer group) and in Fig. 3 (low and intermediate titer groups). Titer courses varied between individual patients, most patients (18/22, 82%) developed their peak antibody titer within the first 12 months of ERT, after which the antibody titer declined and remained stable. Only one patient in the high titer group (patient 2) showed persistent high antibody titers (1:31,250) at all of the time points after the baseline measurement. Antibody titers declined in all the other patients, only patient 1 showed re-occurrence of high antibody titers (1:31,250) after 5 years of treatment. Two patients (patient 4 and 5) showed a high titer at only one time point and in only one of the duplicate experiments (Additional file 1: Figure S1). None of the patients receiving a higher than standard dose of alglucosidase alfa (i.e. > 20 mg/kg/2 weeks, patient 7, 8, 11, 13 and 22) showed high antibody titers. There was no correlation between age at start of ERT and peak antibody titer (Additional file 1: Figure S2).

### Effect of GAA variant/activity on antibody formation

**Fig. 4:**
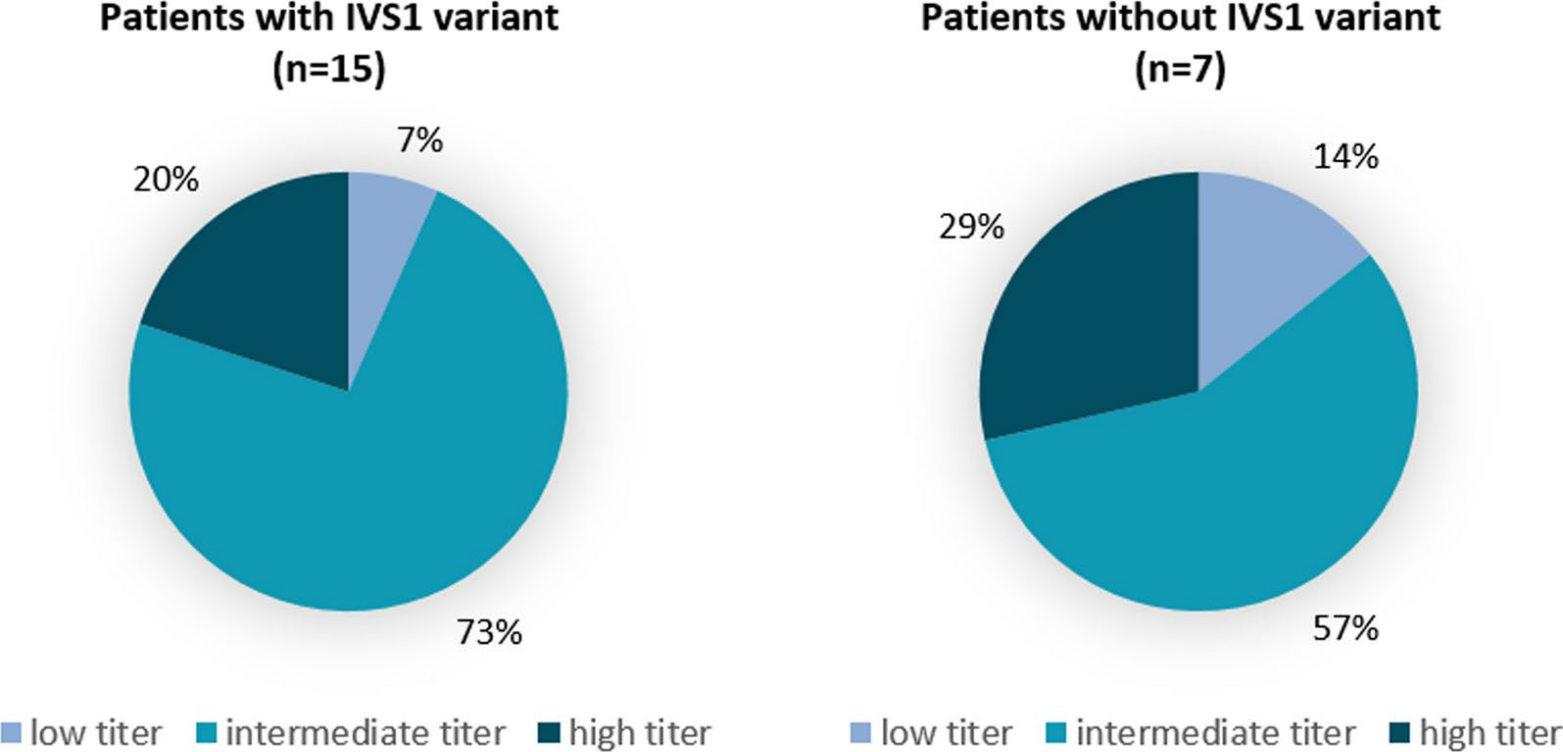
Peak anti-recombinant human acid α-glucosidase (anti-rhGAA) antibody titers in relation to genotype. Pie charts of the distribution of antibody titer groups across patients with (n = 15) and without (n = 7) the c.-32-13 T > G (IVS1) variant

Of the five patients with a high peak titer, three were carrier of the common IVS1 variant on one allele and two carried other pathogenic variants (Table 1). Of the three patients with a high titer at more than one time point, one was carrier of the IVS1 variant (patient 2), one carried a potentially mild variant (c.875A > G) and an unknown variant (patient 3) and one (patient 1) was carrier of one severe variant (c.2481 + 102_2646 + 31del) and one potentially less severe variant (c.2102T > C) [21]. The c.875A > G variant is a potentially mild variant leading to an amino acid substitution with no effect on splicing. The c.2481 + 102_2646 + 31del variant on the other hand, is a very severe variant, leading to an in frame skip of exon 18 and as a consequence an abnormal GAA protein (www.pompevariantdatabase.nl) [21], whereas the c.2102T > C variant was predicted to be a potentially less severe, missense variant (p.Leu701Gln, own prediction). The distribution of peak antibody titers did not differ between patients with/without the IVS1 variant (Fig. 4). The IVS1 variant affects pre-mRNA splicing and induces full or partial skipping of exon 2 of *GAA* as well as utilization of a pseudo exon in intron 1. It allows a low level of (10–15%) of leaky wild type splicing [18]. Of the seven patients with the c.510C > T genetic modifier on the IVS1 allele, one patient had a low peak titer, five patients had an intermediate peak titer and one patient developed a high peak titer [18]. The patient with the highest titer (1:156,250) was carrier of one severe variant and one potentially less severe variant. We did not find a significant correlation between residual (endogenous) α-glucosidase activity in fibroblasts and peak antibody titer (Additional file 1: Table S1 and Figure S3).

### Infusion associated reactions

**Fig. 5:**
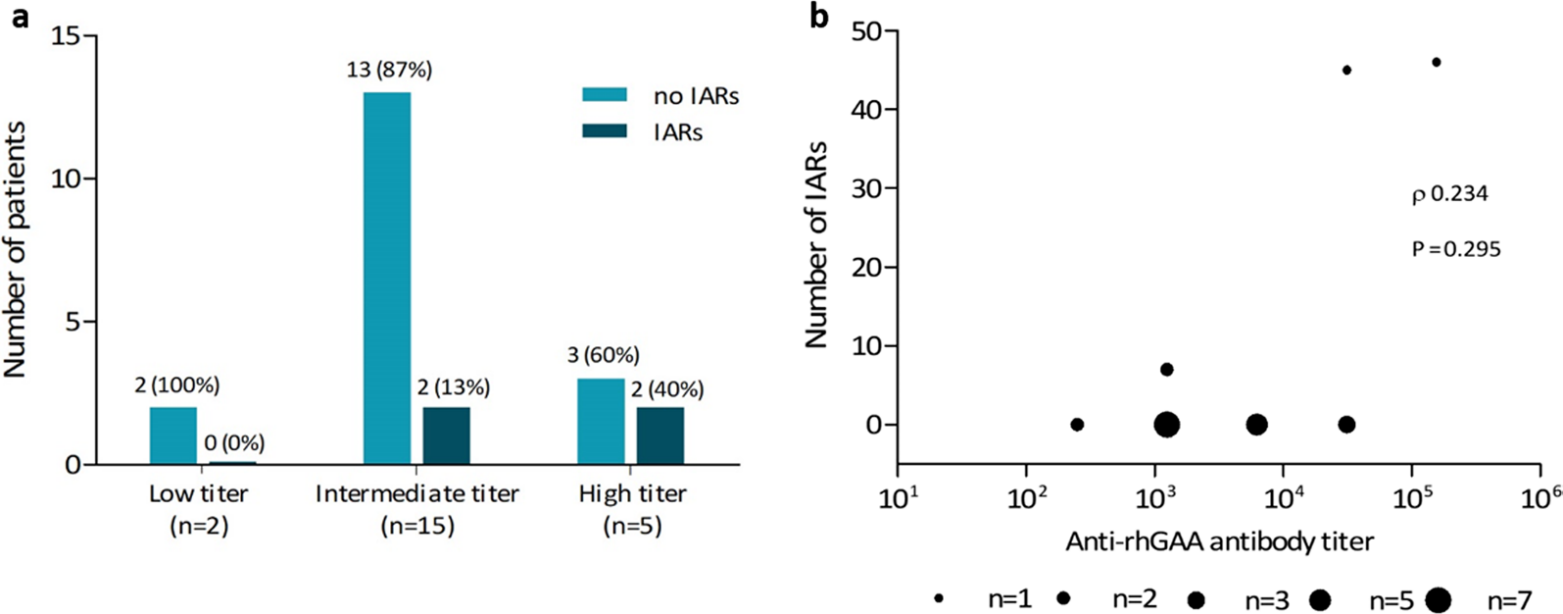
Infusion associated reactions (IARs). **a** The number of patients with and without infusion associated reactions (IARs) across the three anti-recombinant human acid α-glucosidase (anti-rhGAA) antibody titer groups. **b** Correlation between the number of IARs and peak anti-rhGAA antibody titers (Spearman ρ = 0.234, *p* = 0.295)

Four patients (18%) developed IARs during treatment; the distribution of the occurrence of IARs between the titer groups is shown in Fig. 5a. In most patients, the first IAR occurred within the first year of treatment (median onset of IARs after start of ERT 0.21 years, range 0.11–1.15 years). Infusion associated reactions occurred in 59 infusions out of a total of approximately 6200 infusions administered over the years. All IARs occurred during the infusion or within 2 h after completion of the infusion. Types and frequencies of IAR symptoms are summarized in Additional file 1: Table S2. Most common symptoms were nausea, dizziness and exanthema. In general, IAR symptoms were mild and 58 of 59 of infusions (98%) in which IARs occurred could be completed on the same day, after a short interruption of the infusion (n = 24) or an interruption of the infusion plus administration of antihistamines (n = 15). In 13/15 (87%) of the infusions in which antihistamines had to be administered it concerned patients from the high titer group (n = 11 in patient 1 and n = 2 in patient 3). In 20 infusions with IARs, no specific action to complete the infusion had to be undertaken. Only one infusion had to be discontinued, due to more severe symptoms (generalized urticaria, coughing and sweating), which did not completely resolve after administration of antihistamines. Next infusions in this patient were given with premedication (clemastine) and adjusted infusion rates and could be fully completed, although localized urticaria (on arms/legs) occurred in two more infusions in the two months following the first IAR. The patient with the persistent high antibody titer (patient 2) did not develop any IARs. The patient with the highest peak titer (patient 1) showed the largest number of infusions with IARs (n = 28). Overall, the correlation between the number of IARs and the peak antibody titer was not statistically significant (Spearman ρ = 0.234, *p* = 0.295; Fig. 5b). No patients had to discontinue ERT permanently because of (severe) IARs and no patients experienced IARs at the end of the study.

### Neutralizing effects of anti-rhGAA antibodies

Our previous studies in classic infantile and adult Pompe patients showed that the threshold titer at which neutralizing effects may be observed is 1:31,250 [12, 14]. For all five patients in the high peak titer group, at least one of the high titer samples was analyzed for neutralizing effects in vitro. In total, seven of the twelve samples with a titer of ≥ 1:31,250 were analyzed: six samples with an antibody titer of 1:31,250 and one sample with a titer of 1:156,250. Intracellular rhGAA activity in the analyzed samples, which reflects the combined effects of antibodies on cellular uptake and enzyme activity, is shown in Fig. 2. Inhibited enzyme activity of 58% in cells was observed in only one sample of patient 3 at time point t = 6 months (antibody titer 1:31,250). In the other samples, intracellular rhGAA activity was not inhibited, not even in the sample with the highest titer. None of the analyzed samples showed inhibited enzyme activity in medium (data not shown).

### Effect of antibodies on clinical outcome

Clinical course (MRC sum score, QMFT sum score, FVC in upright position) of the five patients in the high peak titer group was analyzed on an individual basis (Fig. 2). Patient 2, 4 and 10 showed a stable course of clinical outcome measures throughout the study. Patient 3 was already very severely affected at start of ERT (completely wheelchair dependent and dependent on invasive ventilation); therefore, only assessment of MRC scores was possible, which initially showed improvement, but further deterioration after 2 years of treatment. Patient 1 was only 1 year old when ERT was started, therefore assessment of the MRC, QMFT and FVC could not be performed during the first years of treatment. The motor domain of the Bayley Scales of Infant Development-II (BSID-II), which is a tool for developmental assessment in the first 2 years of life, showed a delayed motor development at start of ERT (BSID-II SD score <  − 3.0) [22]. During the first 2 years of treatment, the BSID-II improved to SD scores between − 1.7 and − 0.6, which are age-equivalent scores, reflecting normal development (Additional file 1: Table S3). Quick Motor Function Test sum score, MRC sum score and FVC showed a stable or improving course during the study period. In addition, we did not observe a decline in any of the outcome measures when high antibody titers reoccurred after 5 years of treatment. In conclusion, none of the patients showed clear evidence of interference of anti-rhGAA antibodies with ERT efficacy.

## Discussion

In this study, we investigated antibody formation and its possible effects on clinical outcome and the occurrence of IARs during long-term treatment with ERT in childhood onset Pompe disease patients, who also started ERT during childhood. The majority of patients developed anti-rhGAA antibodies above background level, but high peak titers were found in only 23% of patients and a high persistent titer in only one patient. Moreover, only 8% of all samples showed a high titer. We did not observe clear effects of high antibody titers on clinical outcomes, indicating that antibodies do not play a major role in ERT efficacy in childhood onset Pompe disease.

The proportion of patients with a (very) high peak titer in our study is comparable to what we previously reported in adult patients (23% vs. 22%) [14]. We found high sustained antibody titers in only one patient (with short follow up duration), which is also comparable to adults, but in contrast to what is reported in classic infantile Pompe disease patients, especially in CRIM negative patients [13, 22]. We postulated that lower endogenous α-glucosidase activity may lead to higher antibody titers, which was previously hypothesized in adults too [14]. However, we did not find a significant correlation between endogenous α-glucosidase activity and peak titer, although this might (partly) be due to the small number of patients. At an individual level, patients with high peak antibody titers did not have remarkable low α-glucosidase activity compared to patients from the intermediate and low titer groups. Moreover, the patient with the lowest α-glucosidase activity had low peak antibody titers.

Enzyme replacement therapy efficacy was not influenced by high antibody titers in childhood onset patients, not even in the patient with a very high peak titer (1:156,250). However, subtle decline in clinical outcomes may be overlooked, since four of five patients in the high titer group reached maximum scores (100%) on different outcomes at multiple time points (ceiling effect). The patient with a persistent high antibody titer did not show clinical decline, although follow up duration in this patient was only 18 months. Our findings are in contrast to the findings in classic infantile Pompe disease, in which particularly CRIM negative patients are at risk of developing high-sustained antibody titers, which counteract the positive effects of ERT [12, 13]. However, high-sustained antibody titers with negative effects on treatment outcome have also been reported in CRIM positive patients and CRIM status alone does not predict the height or duration of the antibody response [12, 23–25]. Moreover, also adult Pompe patients can develop antibodies against rhGAA that interfere with treatment efficacy [15, 16]. Other factors that have been described to influence antibody formation in classic infantile Pompe patients are dosing of rhGAA and age of treatment initiation [12, 22, 26, 27]. Contrary to what was previously reported, we did not observe development of high antibody titers in any of the patients treated with a higher dose (> 20 mg/kg/every other week) [22, 26]. This applied for patients treated with a higher dose (40 mg/kg/week) from start of treatment, as well as for patients who later switched to a higher dose of rhGAA. In addition, we did not find a relation between age at start of ERT and (peak) antibody titer. However, as age at start of ERT was considerably higher in our patients (all over 1 year of age) compared to the classic infantile patients in previous studies, which reported that patients who started ERT within the first two months of life developed lower titers, the comparison of these patient groups might not be valid [12, 27].

Most patients reached their peak antibody titer within the first year of treatment. This is similar to what we found in our adult cohort and is in line with the reported decrease of antibodies and absence of neutralizing antibodies after long-term exposure to rhGAA [14, 17]. Two of the five patients with a high peak titer showed a high titer (of 1:31,250) at only one time point and only in one of the two experiments, the other experiment showed a titer of 1:6250 (intermediate titer). Differences between the two experiments are caused by the five-fold dilution series used to determine titers, which can result in a titer closest to either 1:6250 or 1:31,250 and thus indicates that the actual titer lies between 1:6250 and 1:31,250. Importantly, these patients showed a stable disease course and never experienced IARs.

Only one patient showed inhibited intracellular rhGAA activity, in one sample with an antibody titer of 1:31,500 at 3 months of treatment. Although this patient was already diagnosed at an early age (symptom onset at the age of 1 year; hypertrophic cardiomyopathy), unfortunately ERT could not be started until the age of 12 years, when ERT was registered as treatment for Pompe disease. Consequently, this patient was already very severely affected (completely wheelchair dependent and invasively ventilated) at start of treatment. Because of further deterioration despite ERT, ERT was discontinued at the age of 22. The patient deceased almost eight months thereafter, at the age of 23. The assessment of clinical outcome measures was complicated by the fact that only manual muscle testing (MRC) could be performed at limited time points. However, the hypertrophic cardiomyopathy resolved after start of ERT and antibody titers declined to intermediate titers after 12 months of treatment. A potential neutralizing effect was only found at one time point, 1 year before clinical deterioration occurred. It can not be ruled out that the neutralizing antibodies contributed to the observed clinical deterioration to some extent in this patient. The other patients with high antibody titers did not show inhibited intracellular GAA activity. Based on the data we present in this study, we postulate that neutralizing antibodies do not play a major role in the efficacy of ERT in childhood onset Pompe disease patients.

In four patients (18%) IARs occurred during treatment, which is a similar percentage to what has been reported in adult patients [14, 28–31]. Eighty-nine percent of the IARs could be managed by a short interruption of the infusion, slowing the infusion rate and/or administration of antihistamines. None of the patients had to discontinue ERT permanently due to IARs. Contrary to what we previously reported in adult patients, we did not find a statistically significant correlation between the peak antibody titer and number of IARs in our patients [14]. This might be due to the small number of patients with IARs, since it is remarkable that the patient with the highest peak antibody titer showed the largest number of IARs and experienced more severe IARs. Moreover, the majority of the total number of IARs (86%) was observed in patients in the high titer group. Thus, there may be an association between high antibody titers and the occurrence of IARs in children as well.

With regard to a possible association between genotype and antibody titers, we did not find any clear associations. However, this could be due to the small number of patients and broad variation of different pathogenic variants among these patients. We found that the patient with the highest antibody titer was not a carrier of the IVS1 variant, whereas most other patients were.

Although high sustained antibody titers do not seem to play a major role in late onset Pompe disease, personalized immunogenicity risk assessment (PIMA) could be a helpful tool to identify patients who are at risk for developing high antibody titers [32]. With PIMA, the risk of developing antibodies (in CRIM positive Pompe disease patients) is predicted by using information about a patient’s native GAA gene and their HLA DR haplotype. This tool is developed using data of infantile onset Pompe disease patients and it predicts the anti-drug antibody status accurately in 64% of patients. To use this tool more broadly, it should first be validated for use in late-onset Pompe disease patients and the accuracy of the model should be refined further. If the tool proofs to be valid in late-onset Pompe disease too, it could be used to differentiate high-risk from low-risk patients and identify patients in whom antibody titers should be monitored more closely.

The most important limitation of our study is the small patient population, inherent to rare diseases. Additionally, we only found potential neutralizing antibodies in one sample of a severely affected patient, with limited availability of clinical data. Therefore, the effect of neutralizing antibodies on clinical outcome remains somewhat uncertain. To draw more robust conclusions, future studies should aim to increase patient numbers, for which international collaboration is needed.

## Conclusions

We conclude that the majority of patients with childhood onset Pompe disease do not develop high antibody titers. High antibody titers do not seem to affect clinical outcome, but may be associated with the occurrence of IARs. Although we did not observe clinical deterioration in our patients with a high peak antibody titer, interference of high antibody titers (with neutralizing effects) with treatment efficacy has previously been reported in late-onset Pompe disease patients [15, 16]. We therefore suggest that antibody titers should be determined in case of (unexpected) clinical deterioration, especially when this decline is accompanied by IARs. If high antibody titers with neutralizing effects are found and judged to be the causative factor for the observed clinical deterioration, immunomodulation, which is used to counteract antibody formation in classic infantile Pompe disease patients, could be considered in late-onset patients as well [22, 23, 25, 33].

## Methods

### Population and study design

We included patients with childhood onset Pompe disease in whom ERT had been started before the age of 18 years. The diagnosis was confirmed by diagnostic enzyme analysis in leukocytes and/or fibroblasts and by mutation analysis. Patients with classic infantile Pompe disease were excluded. Classic infantile Pompe disease was defined as symptoms of (a) muscle weakness within 6 months after birth, (b) hypertrophic cardiomyopathy, (c) less than 1% α-glucosidase activity in fibroblasts, and (d) severe mutations in both GAA alleles. The study was conducted at the Center for Lysosomal and Metabolic Diseases, Erasmus MC University Medical Center in Rotterdam. This is the single reference center for Pompe patients in the Netherlands. Since also patients from outside the Netherlands have been treated and followed in our center as part of several clinical studies, this study includes 16 patients from the Netherlands, two from Belgium, two from Germany, and one patient from the UK and one from the USA [19, 29, 34].

Clinical assessments were performed every 3–6 months by specialized physical therapists and clinicians. Data were collected prospectively from June 1999 to January 2019. Before market approval in 2006, ERT was given as part of trials [19, 29, 34]. Infusions were given in standardized infusion schedules (Additional file 1: Table S4). In case of IARs, infusion schedules were adapted at the discretion of the treating physician. All patients and/or their parents provided written informed consent.

### Antibodies and neutralizing effects

For all patients, anti-rhGAA IgG antibody titers were determined at standard time points: before start of ERT (t = 0) and during ERT at 3, 6 and 12 months and at 3, 5, 7, 11, 15 and 19 years (if applicable). In addition, the sample drawn at the patient’s last visit was analyzed. For two patients, no samples before start of ERT were available. If high antibody titers were found, additional samples at intermediate time points were tested. In total, 152 samples were analyzed. Blood samples were drawn before the infusion or, for patients receiving treatment at home, on a day when patients did not receive ERT. If no sample was available at the before mentioned standard time point, the sample closest to the time point was used.

Antibody titers were determined in plasma or serum using ELISA with fivefold dilution series, as previously described [12, 14]. Experiments were performed in duplicate and repeated at least two times for high titers. The highest titer measured per sample was reported and used for analysis. On each plate, a positive control (i.e. rabbit antiserum) and a negative control (serum from a mix of healthy persons) were included.

For five patients participating in an open-label study, ELISA results obtained during this study are reported for time point 3 months (patient 15), 3 and 6 months (patient 9, 13 and 14) and 3, 6 and 12 months (patient 6) [34]. For patient 2, antibody titers obtained during the randomized controlled Late-Onset Treatment Study (LOTS) are also reported [29]. Antibody titers cutoffs were based on data derived from classic infantile and adult cases: no-low titer (< 1:1250), intermediate titer (1:1250 to < 1:31,250) and high titers (≥ 1:31,250) [12, 14].

The effects of antibodies on the uptake of rhGAA (neutralizing effects) were determined by adding an amount equivalent to 200 nmol 4-Methylumbelliferyl-α-D-glucopyranoside monohydrate (MUGlc)/h alglucosidase alfa per 200 μL medium and 20 μL of the patients’ serum to the medium of α-glucosidase deficient fibroblasts, followed by measurement of α-glucosidase activity in cells and medium, as described before [12, 14]. The experiment was performed in duplicate and repeated if needed. Fibroblasts and medium were harvested after 24 h. Alpha-glucosidase activity was expressed as a percentage of the activity present in medium containing fetal calf serum and measured 24 h after addition of rhGAA to the medium. We defined a neutralizing effect as a diminished enzyme activity both intracellularly and in medium. A potential neutralizing effect was defined as a diminished enzyme activity intracellular only; this can be evidence of neutralizing antibodies or of a decreased cellular uptake of the enzyme.

### Infusion-associated reactions and clinical outcome measures

If an IAR occurred during or following an rhGAA infusion, IAR symptoms and treatment were recorded. Only IARs judged to have a possible, probable or definite relationship with the infusion were included in this study.

Skeletal muscle strength was measured by manual muscle testing using the Medical Research Council (MRC) grading scale (range 0–5) [35]. Muscle groups tested comprised the neck flexors, neck extensors, shoulder abductors, elbow flexors, elbow extensors, wrist extensors, hip flexors, hip extensors, hip abductors, hip adductors, knee flexors, knee extensors, foot dorsal flexors, and foot plantar flexors.

Muscle function was assessed using the Quick Motor Function Test (QMFT), which evaluates 16 motor skills that are specifically challenging for patients with Pompe disease [36]. For the MRC and QMFT, sum scores were derived as described previously [37]. If values for two or more items were missing, no sum score was calculated.

Forced vital capacity (FVC) was measured in upright and supine positions according to ATS/ERS standards [38]. Results were expressed as a percentage of the predicted normal values [39].

### Statistical analysis

We used descriptive statistics (including median and range) to summarize demographic data. For correlations, Spearman’s rho test was used. Because of limited statistical power due to small group sizes, effects of antibodies on clinical outcome measures were described on an individual basis. Statistical analyses were performed using SPSS for Windows (version 25; SPSS Inc, Chicago, IL) and GraphPad Prism (version 5.01 for Windows, GraphPad Software, La Jolla California USA). A *p*-value of < 0.05 (two-sided) was considered statistically significant.

## Supplementary Information

**Additional file 1. Table S1-S4 and Figure S1-S3. Table S1** - Residual α-glucosidase activity. **Table S2** - Infusion Associated Reactions (IARs). **Table S3** - Scores on the motor domain of the Bayley scales of infant development-II for Patient 1. **Table S4** - Standardized infusion protocol. **Figure S1** - Anti-recombinant human acid α-glucosidase (anti-rhGAA) antibody titer course in Experiment 1 and Experiment 2. **Figure S2** - Correlation between the age at start of ERT and peak anti-rhGAA antibody titers. **Figure S3** - Correlation between residual α-glucosidase activity and peak anti-rhGAA antibody titers.

## Data Availability

The corresponding author takes full responsibility for the data, has full access to all data and has the right to publish all data apart from any sponsor. Anonymized data will be shared by request from any qualified investigator for the sole purpose of replicating procedures and results presented in the article, and as long as data transfer is in agreement with EU legislation on the general data protection regulation.
