# Supplementary material for: Antibodies against recombinant human alpha-glucosidase do not seem to affect clinical outcome in childhood onset Pompe disease
Source: Orphanet J Rare Dis. 2022 Feb 2;17:31. doi: 10.1186/s13023-022-02175-2 (PMC8812154; doi:10.1186/s13023-022-02175-2)
Supplement: Supplementary file 1 — Additional file 1. Table S1-S4 and Figure S1-S3. Table S1 - Residual α-glucosidase activity. Table S2 - Infusion Associated Reactions (IARs). Table S3 - Scores on the motor domain of the Bayley scales of infant development-II for Patient 1. Table S4 - Standardized infusion protocol. Figure S1 - Anti-recombinant human acid α-glucosidase (anti-rhGAA) antibody titer course in Experiment 1 and Experiment 2. Figure S2 - Correlation between the age at start of ERT and peak anti-rhGAA antibody titers. Figure S3 - Correlation between residual α-glucosidase activity and peak anti-rhGAA antibody titers. [file 13023_2022_2175_MOESM1_ESM.docx]

**Additional file 1**

**Table S1 – Residual α-glucosidase activity**

| Patient | α-glucosidase activity  in cultured fibroblasts (nmol/h/mg) |
| --- | --- |
| 1 | NA |
| 2 | 7.8 |
| 3 | NA |
| 4 | 11.9 |
| 5 | 15.4 |
| 6 | 8.6 |
| 7 | 3.4 |
| 8 | 9.1 |
| 9 | 8.4 |
| 10 | 8.7 |
| 11 | 0.7 |
| 12 | 11.0 |
| 13 | 2.8 |
| 14 | 17.9 |
| 15 | 13.3 |
| 16 | 13.0 |
| 17 | NA |
| 18 | 6.2 |
| 19 | NA |
| 20 | 11.6 |
| 21 | 6.0 |
| 22 | 0.4 |

nmol/h/mg: nanomol per hour per milligram; NA: data not available

**Table S2 - Infusion Associated Reactions (IARs)**

| IAR symptoms | Number of IARs (%) | Number of patients with IAR (%)* |
| --- | --- | --- |
| Nausea | 23 (21.6) | 2 (50) |
| Dizziness | 15 (14.1) | 2 (50) |
| Exanthema (localized) | 11 (10.4) | 2 (50) |
| Urticaria (localized) | 10 (9.4) | 3 (75) |
| Urticaria (generalized) | 10 (9.4) | 2 (50) |
| Itching (localized) | 7 (6.6) | 1 (25) |
| Coughing | 6 (5.7) | 2 (50) |
| Itching (generalized) | 6 (5.7) | 1 (25) |
| General malaise | 5 (4.7) | 1 (25) |
| Sweating | 4 (3.8) | 2 (50) |
| Exanthema (generalized) | 3 (2.8) | 3 (75) |
| Hyperthermia | 2 (1.9) | 2 (50) |
| Dyspnea | 2 (1.9) | 1 (25) |
| Headache | 1 (0.9) | 1 (25) |
| Local angioedema | 1 (0.9) | 1 (25) |
|  |  |  |
| Total | 106 (100) | 4 (100) |
| Total infusions with IARs | 59 |  |

IAR: infusion associated reaction; * patients may have experienced more than one symptom during the course of an IAR.

**Table S3 - Scores on the motor domain of the Bayley scales of infant development-II for patient 1**

| Time from start of ERT (years) | Raw score | Development index | SD-score |
| --- | --- | --- | --- |
| 0.23 | 60 | <55 | <-3.0 |
| 0.54 | 78 | 60 | -0.7 |
| 1.0 | 89 | 75 | -1.7 |
| 1.26 | 97 | 87 | -0.9 |
| 1.53 | 98 | 81 | -1.3 |
| 1.76 | 102 | 91 | -0.6 |

ERT: enzyme replacement therapy; SD: standard deviation.

**Table S4 - Standardized infusion protocol**

|  | Speed of infusion (ml/h) | Duration (min) |
| --- | --- | --- |
| Step 1 | 5 | 30 |
| Step 2 | 20 | 30 |
| Step 3 | 88 | 30 |
| Step 4 | 250 | Rest |

ml/h: milliliters per hour; min: minutes.


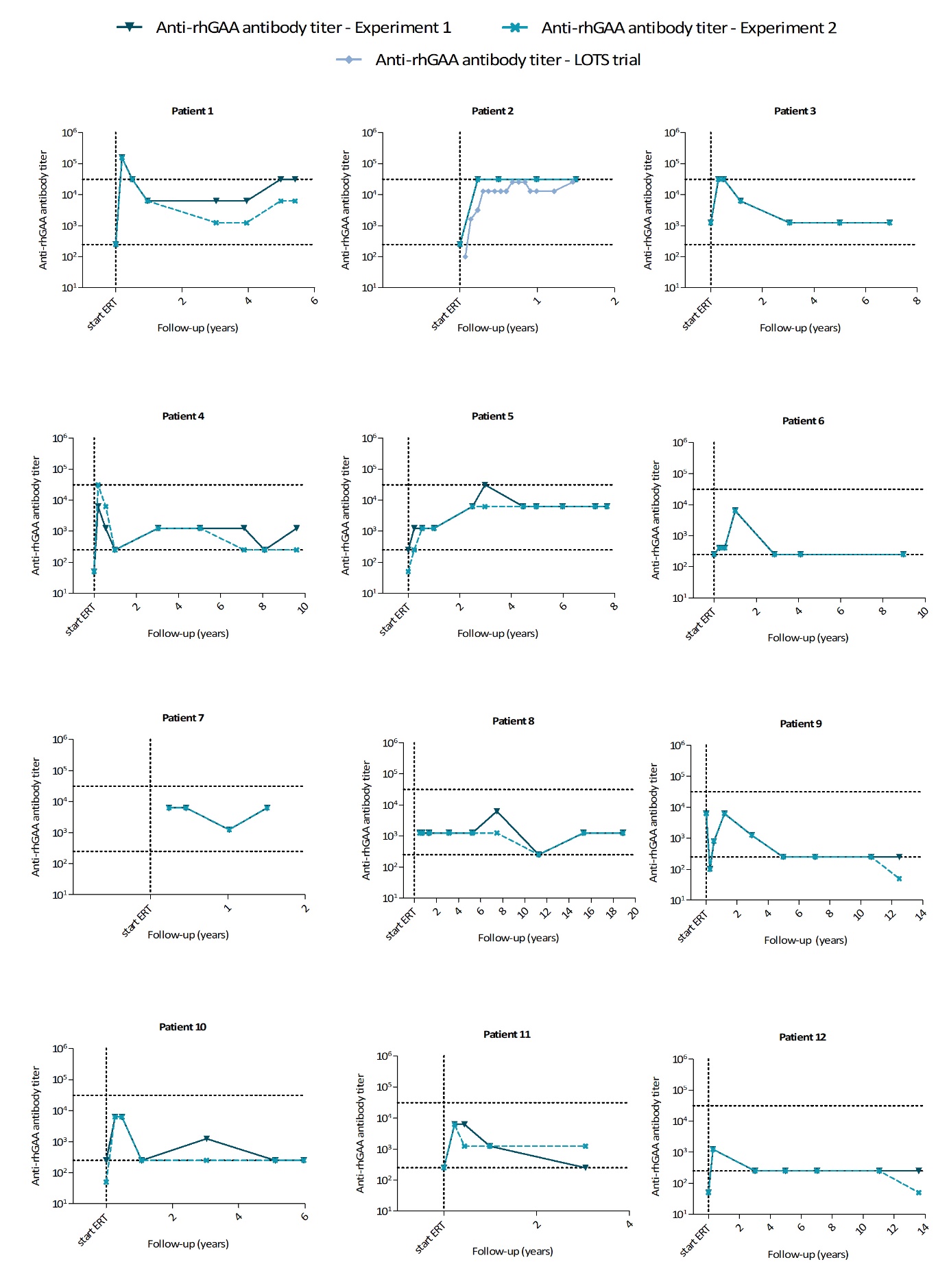


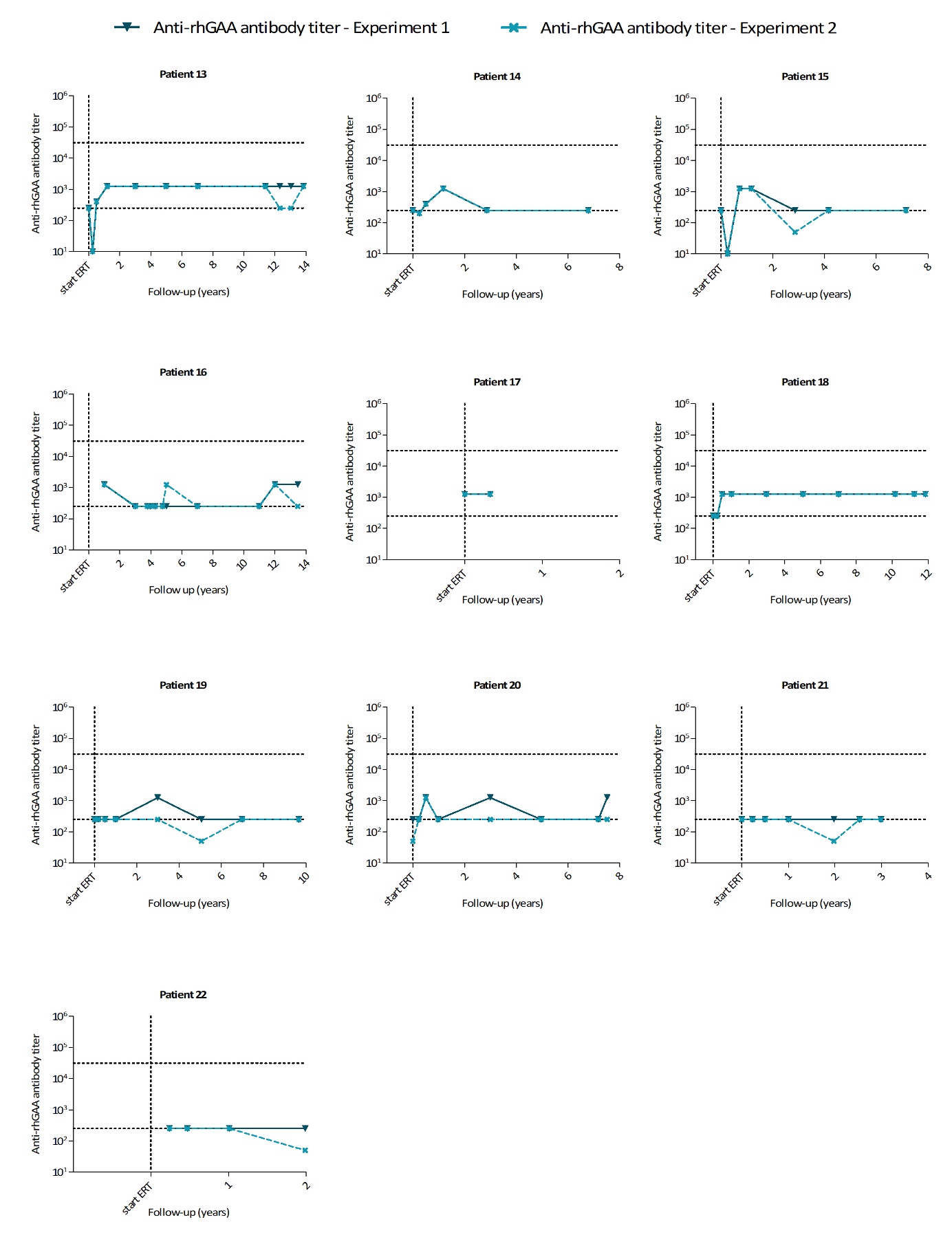


**Figure S1 - Anti-recombinant human acid α-glucosidase (anti-rhGAA) antibody titer course in Experiment 1 and Experiment 2.** Each graph represents an individual patient. For each patient, the anti-rhGAA antibody titer measured in Experiment 1 and Experiment 2 are shown. For patient 2, anti-rhGAA antibody titers obtained during the Late-Onset Treatment Study (LOTS, van der Ploeg 2010) are shown as well.

**
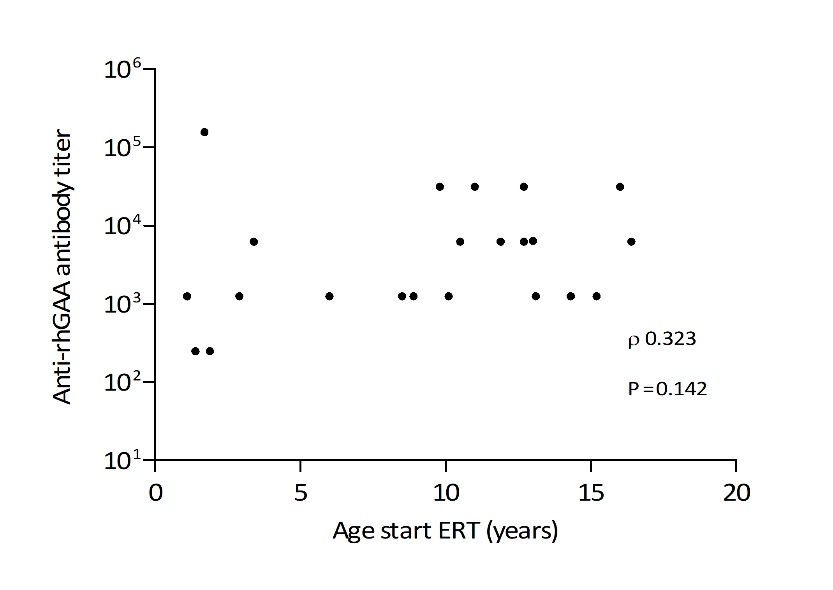
**

**Figure S2 – Correlation between the age at start of ERT and peak anti-rhGAA antibody titers.** Correlation analysis showed no significant correlation (Spearman ρ = 0.323, p=0.142).


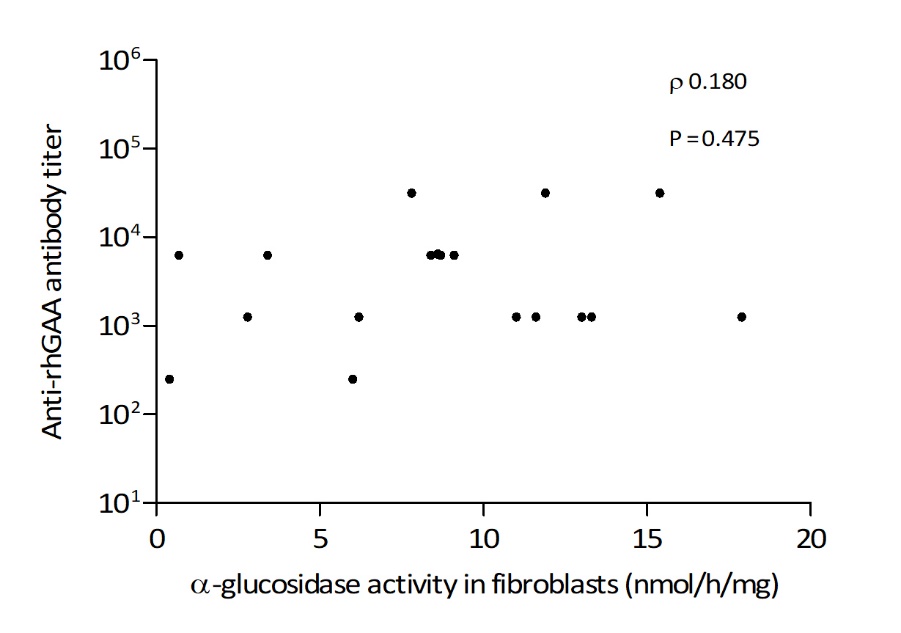


**Figure S3 Correlation between residual a-glucosidase activity and peak anti-rhGAA antibody titers.** Correlation analysis showed no significant correlation (Spearman ρ = 0.180, p=0.475).
